# Pre-treatment with EDTA-gallium prevents the formation of biofilms on surfaces

**DOI:** 10.3892/etm.2013.946

**Published:** 2013-02-05

**Authors:** YUANYUAN ZHU, FENG JIN, SHUANGWANG YANG, JINGNONG LI, DAN HU, LIANMING LIAO

**Affiliations:** 1 Nanjing Junxie Hospital, Nanjing, Jiangsu;; 2 476 Clinical Department, Fuzhou General Hospital, Xiamen University, Fuzhou, Fujian, P.R. China; 3 Academy of Integrative Medicine, Fujian University of Traditional Chinese Medicine, Fuzhou, Fujian, P.R. China

**Keywords:** gallium, biofilm

## Abstract

*Pseudomonas aeruginosa* and *Streptococcus pyogenes* are leading causes of medical device-associated infections. The capacity to establish and maintain these infections is thought to be associated with the ability to form surface-attached biofilms. In the present study, gallium nitrate was used to coat PVC plates and biofilm formation on the plates by *Pseudomonas aeruginosa* and *Streptococcus pyogenes* was evaluated. The results demonstrated that coating the PVC surface with gallium reduced bacteria cell aggregation on the PVC surface and inhibited biofilm formation. These results suggest that surface pre-treatment with a gallium nitrate coating is a potential strategy for the prevention of infections associated with *Pseudomonas aeruginosa* or *Streptococcus pyogenes* on medical devices.

## Introduction

Biofilms are surface-attached colonies of bacteria on various biotic and abiotic surfaces, encased in a hydrated matrix of exopolymeric substances, proteins, polysaccharides and nucleic acids. Biofilms play a major role in the pathogenesis of device-associated infections. Biofilm formation is a complex developmental process (1–4). Surface-attached bacterial biofilms are considered to be a significant source of nosocomial infections, which are responsible for intravascular device-associated bacteremias and ventilator-associated pneumonia (5–7). Biofilm formation allows for immune evasion and resistance to antibiotics since the bacteria in a biofilm are protected by a matrix, and the slow rate of metabolism of cells enables them to survive 100- to 1000-fold concentrations of antibiotics (8–10). Heavy antibiotic use has greatly increased antibiotic resistance due to genetic mutation and this problem is continually increasing in severity (10,11). Recently, much attention has been focused on the need for new antimicrobial agents (12–16).

For the majority of pathogens, iron (Fe) is essential for growth and the functioning of key enzymes, including those involved in DNA synthesis, electron transport and oxidative stress defense (17). The post-transition metal gallium (Ga) has an ionic radius almost identical to that of Fe, and numerous organisms are unable to distinguish Ga^3+^ from Fe^3+^. Ga is able to disrupt Fe-dependent processes since, unlike Fe^3+^, Ga^3+^ cannot be reduced under physiological conditions, and sequential oxidation and reduction are critical for many of Fe’s biological functions. *In vitro* and *in vivo* studies show that Ga^3+^ inhibits the growth and biofilm formation of various pathogens by interfering with Fe signaling (18).

Numerous polymers, such as polyvinyl chloride (PVC), are widely used biomaterials for cardiovascular and other artificial devices. In this study, we developed a new Ga-based coating material suitable for PVC. We demonstrated that following the surface treatment of PVC with a Ga coating, biofilm formation may be inhibited.

## Materials and methods

### Bacterial strains and culture

*Pseudomonas aeruginosa* was cultured in LB liquid medium (consisting of 10.0 g/l peptone, 5.0 g/l yeast extract and 10.0 g/l NaCl, pH 7.0–7.2) and incubated at 37°C with shaking (180 rpm) for 24 h. The inoculum was collected and diluted with normal saline.

*Streptococcus pyogenes* was cultured in Müller-Hinton (MH) medium supplemented with 10% (v/v) sheep blood and incubated at 36°C with shaking (120 rpm) for 6 h. The inoculum was collected and diluted with normal saline.

### Minimum inhibitory concentration (MIC) determination

Overnight cultures (37°C) of the tested strains were diluted 10-fold in fresh tryptic soy broth (TSB) and incubated (37°C) until the exponential growth phase was reached. The inocula (10 *μ*l) containing 5×10^6^ CFU/ml of each reference strain were added to each well in the presence or absence of gallium nitrate at different concentrations. Certain wells were reserved in each plate to test the sterility control of the medium (no inoculum added) and inoculum viability (no gallium nitrate added). The turbidity of the medium was directly proportional to the growth of the bacteria, which was measured by a microtiter plate reader (Tecan, Milan, Italy) at 600 nm absorbance. After incubation for 24 h at 37°C, non-adherent bacteria were washed three times with 0.9% (w/v) NaCl. The biofilms were stained with 0.1% (w/v) crystal violet solution for 10 min, washed and then dissolved with 33% (v/v) acetic acid for 10 min. The biofilm mass was determined by measuring the absorbance at 590 nm using a microtiter plate reader. The MIC was defined as the concentration that completely inhibited visible cell growth during a 24-h incubation period at 37°C.

### Surface coating

The gelatin aqueous solution (0.5%) was auto-claved. After cooling, 50 *μ*l gelatin solution was mixed with gallium nitrate (Sigma, Bornem, Belgium) and a stock solution of a chelator. The final concentrations of gallium nitrate and various chelators are shown in Table I. The mixture was added to the PVC 96-well plate and left to dry at 37°C in a sterile incubator overnight. The wells were then washed using saline to remove loosely attached Ga from the surface of the wells.

### Antimicrobial durability

The antimicrobial efficacy of the Ga-coated PVC chip was assessed over time in a system that imitated the blood stream flushing through the surface of the endovascular medical device. PVC chips of identical surface area were dipped into the Ga-chelator-gelatin solution and allowed to dry twice. The chips were then placed in a container through which water flowed at a speed of 2 cm/sec. After 3 days, the amount of Ga remaining on the Ga-coated PVC chip was measured and its antimicrobial efficacy was evaluated.

The inhibitory index of the Ga coat=[1–(absorbance of the biofilm on the assay chips/absorbance of the biofilm on the negative control chips)]×100%.

To determine the amount of Ga remaining after washing, the PVC chips were burned to ash in a crucible. The content of Ga in the ash was analyzed by back titration with a standard zinc solution.

### Analysis of the Ga cation content by back titration with a standard zinc solution

The coated PVC chips treated with the flow-erosion system were burned to ash in a crucible. The ash was dissolved in aqueous hydrochloric acid solution (2 M) over heat. The solution was then transferred into a volumetric flask and the volume was standardized to 50 ml and homogenized well. An excess of ethylenediamine-N,N,N′,N′-tetraacetic acid (EDTA) solution was added to 10 ml of the solution and the pH was adjusted to 5.8 with hexamethylenetetramine buffer. Xylenol orange (5 drops) was added as an indicator.

A zinc standard solution was used for back titration to determine the Ga content. The PVC chips not subjected to water erosion were used as a positive control.

### Static biofilm formation

A static biofilm formation assay was carried out in 24-well PVC microtiter plates (Falcon; Becton Dickinson Labware, Oxnard, CA, USA). Briefly, overnight bacteria cultures were diluted to ∼1×10^6^ CFU/ml with fresh sterile medium. Aliquots (100 *μ*l) of the diluted cultures were added to each well pre-coated with gelatin in the presence of different concentrations of Ga. The plates were incubated at 37°C for 24 h without agitation for biofilm growth and non-adherent bacteria were washed away three times with 0.9% (w/v) NaCl. The biofilms were strained with 0.1% (w/v) crystal violet solution for 10 min and washed three times. Adherent bacterial cells were observed by phase contrast microscopy.

Isopropanol (150 *μ*l)-0.04 M HCl and 50 ml of 0.25% SDS were added to each well to resolubilize crystal violet. The biofilm mass was determined by measuring the absorbance at 590 nm using the microtiter plate reader. Assays were carried out three times in five replicates.

### Statistical analysis

Statistical analysis was performed using a paired Student’s t-test, with Bonferroni correction, or one sample Student’s t-test. P<0.05 was considered to indicate a statistically significant result.

## Results

### Determination of MIC

**Figure 1:**
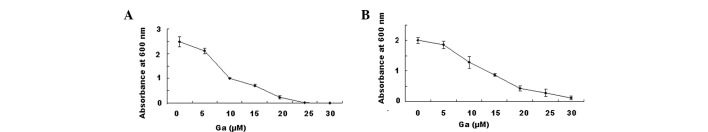
Effect of gallium nitrate on bacterial proliferation. (A) *Pseudomonas aeruginosa* and (B) *Streptococcus pyogenes* were grown in 96-well microplates with medium containing gallium nitrate at concentrations ranging from 5 to 30 *μ*M. The plates were incubated at 37°C for 24 h without agitation. The turbidity of the medium was directly proportional to the growth of the bacteria, which was measured by a microtiter plate reader at 600 nm absorbance.

*Pseudomonas aeruginosa* (Fig. 1A) and *Streptococcus pyogenes* (Fig. 1B) were grown in 96-well microplates with medium containing gallium nitrate at concentrations ranging from 5 to 30 *μ*M. The plates were incubated at 37°C for 24 h with agitation. The turbidity of the medium was directly proportional to the growth of the bacteria, which was measured by a microtiter plate reader at 600 nm absorbance. As shown in Fig. 1, gallium nitrate inhibited the growth of bacteria in a dose-dependent manner, with a MIC of ∼20 *μ*M.

### Effects of different ligands on gallium nitrate adhesion

To select the most effective chelator to prevent Ga cations on the surface of the medical device from being eroded by the blood stream, we fixed Ga cations onto the surface with various ligands. The efficiency was judged by the amount of the Ga remaining in the coat and the antibacterial activity of the remaining Ga coat following the erosion of the PVC chip for 3 days. The Ga coat not subjected to water flow was used as a control to measure the amount of Ga and the antibacterial activity of the Ga coat.

We investigated commercial ligands including EDTA, ethylene glycol-O,O′-bis(2-aminoethyl)-N,N,N′,N′-tetraacetic acid (EGTA), salicylic acid and chloride anion in various ratios with respect to the Ga cation. As demonstrated in Table I, when the concentration was kept at 20 nM, a mixture of EDTA and Ga at a ratio of 2:1 in the gelatine coat effectively inhibited bacterial growth. After flushing for 3 days, 97% of the initial amount of Ga cation remained on the surface and inhibited biofilm formation by 99% compared with that observed on a negative control PVC chip coated with gelatin and EDTA.

When the ratio of EDTA to Ga was >2:1, although 100% Ga remained in the gelatin, the inhibitory index was low.

### Biofilm inhibition

**Figure 2:**
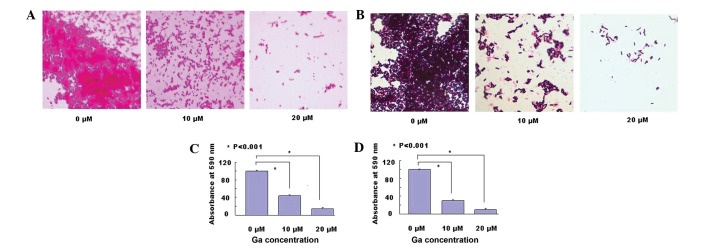
Microscopic views of the biofilm formation after crystal violet staining and quantitation. Biofilms formed by (A) *Pseudomonas aeruginosa* and (B) *Streptococcus pyogenes* were evaluated at different concentrations of gallium nitrate by visualization with crystal violet staining. The amount of biofilm formed by (C) *Pseudomonas aeruginosa* and (D) *Streptococcus pyogenes* was quantitatively measured at 590 nm.

To assess the ability of surface pre-treatment with Ga to inhibit bacterial biofilm formation, *Pseudomonas aeruginosa* or *Streptococcus pyogenes* cell suspensions were placed into Ga-treated plates and cultured at 37°C for 24 h without agitation. Thereafter, the plates were washed and stained with crystal violet, before visualization under a phase contrast microscope. As demonstrated in Fig. 2A and B, a clear decrease of the biofilm with crystal violet staining was observed in the wells pre-treated with Ga.

In addition, after the bacteria were cultured, the unattached bacteria were washed away. The remaining biofilm was stained by crystal violet and dissolved with acetic acid. The biofilm mass was determined by measuring the absorbance at 590 nm using a microtiter plate reader. As Fig. 2C and D demonstrate, the biofilms of *Pseudomonas aeruginosa* and *Streptococcus pyogenes* were reduced by 85% and 90%, respectively, at 20 *μ*M Ga.

## Discussion

An increasing number of clinical procedures require the use of biomedical devices. These devices, used in either the short- or long-term, are associated with device-associated infections due to bacterial colonization and proliferation (19). It is estimated that ∼half of the 2 million cases of nosocomial infections that occur each year in the United States are caused by indwelling devices (20). These infections result in morbidity and mortality of the patients and increased costs for the healthcare system. This is especially true in orthopedic medicine, where biofilms may result in serious complications involving prosthetic and other medical device infections, impacting morbidity, mortality and medical costs (21,22). The bacteria in a biofilm are much more resistant to antibiotics than their planktonic form. Implants increase the risk of infection 100,000-fold, possibly due to the impairment of the microbiocidal activities of granulocytes (23).

Biofilm formation is a complex developmental process involving attachment and immobilization on a surface, cell-to-cell interaction, microcolony formation, formation of a confluent biofilm and development of a three-dimensional biofilm structure. Bacterial biofilms are able to form on either biotic or abiotic surfaces (1–4). The removal of biofilms from the infected site poses a great challenge to the physicians since it usually requires surgical intervention and radical antimicrobial therapy (20).

Due to the resistance of biofilms to antibiotics, new strategies to increase the sensitivity of pathogens in biofilms or new bacteria-killing agents are needed. The replacement of Fe^3+^ with Ga^3+^ interferes with bacterial DNA and protein synthesis, and blocks the redox reactions that depend on Fe electron acquisition. The replacement of Fe has been demonstrated to inhibit *Pseudomonas aeruginosa* growth and biofilm formation and kill planktonic and biofilm bacteria *in vitro*. In the present study, we further evaluated the effects of a Ga-coating on biofilm formation on PVC, a material often used for medical implants. We identified several ligands that were suitable for retaining Ga in a gelatin coat on the surface of a PVC plate. The results indicated that a mixture of EDTA and Ga in a 2:1 ratio in gelatin was the most effective for retaining Ga on the PVC surface. After flushing for 3 days, 97% Ga remained on the surface and biofilm formation was almost completely inhibited. The average velocity of the blood stream in the main veins, including the inferior vena cava, pulmonary vein, portal vein, right femoral vein and left femoral vein, is ∼0.35 cm/sec, and the speed may reach up to 0.8 cm/sec following alcohol consumption. Therefore we used a water-flow speed of 2 cm/sec to erode the coat of the PVC chips.

Ga is already FDA-approved for the treatment of hyper-calcemia of malignancy. As *Pseudomonas aeruginosa* may cause acute and chronic lung infections and result in significant morbidity and mortality in patients with cystic fibrosis, and *Streptococcus pyogenes* is one of the main pathogens responsible for respiratory infections and rheumatic heart disease, an EDTA-Ga-gelatin coating on implants may be a potential strategy in the prevention of infections associated with medical devices.

## Figures and Tables

**Table I:** Compositions of gallium (Ga) and ligands, and inhibition of biofilm formation.

Ligand	Molar ratio (ligand:Ga)	Ga remaining (%)	Inhibitory index (%)
EDTA	1:1	50	41
	2:1	97	99
	3:1	100	70
	4:1	100	43
EGTA	1:1	39	35
	2:1	92	87
	3:1	100	72
	4:1	100	67
EDTA+EGTA	1:1:1	94	89
EDTA+EGTA	1:2:1	100	67
EDTA+salicylic acid	1:1:1	81	77
EDTA+salicylic acid	1:2:1	96	81
EDTA+chloride anion	1:1:1	66	68
EDTA+chloride anion	1:2:1	85	70

EDTA, ethylenediamine-N,N,N′,N′-tetraacetic acid; EGTA, ethylene glycol-O,O′-bis(2-aminoethyl)-N,N,N′,N′-tetraacetic acid.
